# Maternal Haemoglobin and Haematocrit During Pregnancy and Stillbirth Risk: A Prospective, Population‐Based Birth Cohort Study

**DOI:** 10.1111/ppe.70144

**Published:** 2026-03-31

**Authors:** Shuangying Li, Nina Dempsey, Shen Gao, Shaofei Su, Shuanghua Xie, Zhengyang Yao, Minhui Hu, Juan Li, Zhan Li, Enjie Zhang, Jianhui Liu, Hao Xing, Jinghan Yu, Shaowen Wu, Wentao Yue, Ruixia Liu, Alexander E. P. Heazell, Chenghong Yin

**Affiliations:** ^1^ Department of Central Laboratory Beijing Obstetrics and Gynecology Hospital, Capital Medical University. Beijing Maternal and Child Health Care Hospital Beijing China; ^2^ Department of Life Sciences, Faculty of Science and Engineering Manchester Metropolitan University Manchester UK; ^3^ Department of Obstetrics, Beijing Obstetrics and Gynecology Hospital Capital Medical University. Beijing Maternal and Child Health Care Hospital Beijing China; ^4^ Department of Research Management Beijing Obstetrics and Gynecology Hospital, Capital Medical University. Beijing Maternal and Child Health Care Hospital Beijing China; ^5^ Maternal and Fetal Health Research Centre, Division of Developmental Biology and Medicine Faculty of Biology, Medicine and Health, University of Manchester Manchester UK; ^6^ Saint Mary's Hospital, Manchester University NHS Foundation Trust Manchester UK

**Keywords:** anaemia, haematocrit levels, haemodilution, haemoglobin concentrations, stillbirth

## Abstract

**Background:**

Maternal anaemia is a public health issue globally, associated with adverse maternal and neonatal outcomes. However, less is known about the effects of high haemoglobin (Hb) and haematocrit (Hct) on the risk of stillbirth.

**Objectives:**

We assessed the dose–response effects of dynamic changes in maternal Hb and Hct across all trimesters on stillbirth risk.

**Methods:**

This study was based on the prospective, longitudinal, population‐based China birth cohort study (CBCS) between 2018 and 2022, including 142,715 women with live births or stillbirths delivered at ≥ 20 weeks' gestation. The modified Poisson regression models were used to assess the association between Hb/Hct, haemodilution status across three trimesters and stillbirth.

**Results:**

Over a fifth (23.6%) of pregnant women had anaemia at least once during pregnancy. Maternal anaemia increased the risk of stillbirth. The adjusted risk ratio (aRR) for Hb at 70–99 g/L (moderate anaemia) was 2.45, 95% confidence interval (CI) 1.26, 4.76 and aRR for Hct < 33% (anaemia) was 1.63 (95% CI 1.12, 2.38) in the first trimester. Interestingly, a U‐shaped association between Hb/Hct and stillbirth was observed, and high Hb/Hct levels in any of the three trimesters were related to increased stillbirth risk. Excessive haemoglobin dilution/haemoglobin concentration across trimesters was also associated with higher stillbirth risk, especially from the first to the second trimesters, with aRRs of 1.86 (95% CI 1.15, 3.01) and 3.34 (95% CI 2.08, 5.37), respectively.

**Conclusions:**

Considering the U‐shaped association between Hb/Hct levels and stillbirth, clinical vigilance is necessary at both low‐ and high‐extremes of Hb/Hct and physiological haemodilution metrics in mid‐to‐late pregnancy for improved pregnancy outcomes.

## 1 Background

The association between anaemia during pregnancy and adverse perinatal outcomes remains inconclusive [1, 2]. Maternal anaemia affects 36.5% of pregnant women worldwide, with prevalence ranging from 18.9% in the Americas to 47.8% in Southeast Asia [3]. In China, 18.5% of pregnant women were reported to be anaemic in 2019, a similar figure to that in high‐resource countries [3].

Previous studies have linked maternal anaemia to increased risks of preterm birth [4, 5, 6, 7, 8, 9], small for gestational age (SGA) [4, 6, 7, 8], low birthweight (LBW) [6, 7, 9, 10] and stillbirth [4, 5, 6, 8, 11, 12, 13, 14, 15, 16], with stronger associations for moderate to severe anaemia [5, 6, 13, 16]. Interestingly, high haemoglobin (Hb) levels (> 130 g/L or > 145 g/L) were also associated with adverse outcomes [4, 7]. One large prospective, multinational INTERBIO‐21st study revealed a U‐shaped relationship between maternal haemoglobin and unfavourable pregnancy outcomes [7]. Other studies have also shown that increased adverse foetal outcomes occurred at both extremes of Hb and haematocrit (Hct) levels [7, 17, 18, 19, 20, 21]. However, there is a lack of consensus with regard to the stage at which Hb or Hct levels are important in determining pregnancy outcomes; while some studies link early pregnancy anaemia with stillbirth risk, others suggest anaemia in late trimesters is protective [14, 15]. These inconsistencies have been partly attributed to physiological haemodilution among pregnant women.

Haemodilution is a phenomenon in which the expansion in plasma volume is disproportionately greater than the increase in erythrocytes during pregnancy [22]. Plasma volume expands 40%–50% during mid‐pregnancy, out‐pacing red blood cell mass increases (15%–25%), causing Hb to dip in the second trimester before a slight late‐term rise [22]. Impaired haemodilution has been shown to correlate with poor outcomes [7, 10, 11]. Maternal Hct has also been shown to be a key indicator of haemodilution status [23, 24].

This study was conducted to address inconsistent associations between anaemia across all trimesters of pregnancy and stillbirth by analysing data from CBCS. Measuring both Hb and Hct during pregnancy provides a fuller, more reliable assessment of maternal blood status, distinguishing between true anaemia and haemodilution, which is essential for optimal maternal and foetal outcomes. We aimed to evaluate how dynamic fluctuations in maternal blood parameters affect the risk of stillbirth.

## 2 Methods

### 2.1 Data Source and Study Participants

This analysis included data from 18 hospitals participating in the CBCS. The CBCS was a population‐based, prospective, longitudinal cohort study in China to assess risk factors for birth defects and other pregnancy outcomes [25]. The study was performed in mainland China from February 12, 2018, to December 31, 2022 [25]. Details regarding the CBCS have been previously published [25]. Clinical information was collected via questionnaires, including maternal demographic characteristics, residential address, current pregnancy conditions, medication use, pre‐existing diseases, and pregnancy outcomes [25]. Participants in the CBCS were recruited at 6–13^+6^ gestational weeks and followed up at 20–23^+6^, 28–33^+6^ weeks, and after delivery [25]. Identification of participants' data was shown (Figure S1). Participants who withdrew from the study, were unable to complete a baseline questionnaire or were lost to follow‐up were all excluded, as were participants who had a pregnancy loss < 20 weeks, a termination of pregnancy (TOP) at ≥ 20 weeks, an uncertain TOP or stillbirth at ≥ 20 weeks or women without any Hb/Hct available during pregnancy (Figure S1). Therefore, 142,715 women who delivered live births or stillbirths at ≥ 20 weeks' gestation and had at least one Hb or Hct value recorded throughout pregnancy were included (Figure S1).

### 2.2 Exposure

The main exposure variables were the first maternal Hb concentration and Hct values recorded in the first, second and third trimesters, as well as their changes across the three trimesters. Hb/Hct measurements were recommended at 6–13^+6^, 20–24, 25–28 and 29–32 weeks of pregnancy, based on prenatal guidelines in China [26]. We considered Hb and Hct concentrations both as continuous variables and categorical variables. Anaemia and severity levels were determined using the trimester‐specific cutoffs defined by the WHO (2024) and the CDC (1998) [7, 23]. The high Hb and Hct levels were classified based on two previous reports [4, 19]. Considering the effect of residence elevation and smoking status on maternal Hb/Hct levels, observed Hb concentrations (WHO, 2024) and Hct values (CDC, 1998) were adjusted for individuals' altitude and cigarette numbers [7, 23].

We used weekly changes in Hb/Hct levels between trimesters to present the haemodilution status throughout pregnancy. Weekly changes in Hb/Hct levels between trimesters (2nd–1st), (3rd–1st) and (3rd–2nd) were computed as the difference of Hb/Hct levels at the second and first trimesters; the third and first trimesters and the third and second trimesters, divided by the time period in weeks. Measurement periods of less than 3 weeks were excluded from analysis [12, 14]. Weekly changes in Hb/Hct levels during pregnancy were set at more than two standard deviations (SD) below or above the mean, with the middle category as a reference.

### 2.3 Outcomes

Stillbirths are referred to as foetal deaths after 20 weeks' gestation, excluding TOP and uncertain TOP or stillbirth. TOP indicated voluntary induction due to foetal birth defects or serious maternal complications. Stillbirths were also grouped into singleton or multiple pregnancies, preterm or term stillbirths and SGA or non‐SGA stillbirths. Preterm birth was a birth less than 37 weeks. We defined SGA as newborn birth weight below the 10th percentile for a given gestational age and foetal gender using the INTERGROWTH‐21st newborn size standard [27].

### 2.4 Statistical Analysis

We used a Directed Acyclic Graph (DAG) to identify confounders by reviewing previous studies on maternal Hb/Hct levels and stillbirth (Figure S2) [4, 5, 12, 13, 16, 24].

Modified Poisson regression models with robust error estimation were used to estimate risk ratio (RR) and 95% CI for the relationships between Hb or Hct concentration in the first (6–13^+6^ weeks), second (14–27^+6^ weeks) and third (≥ 28 weeks) trimesters and stillbirth. The Hb (or Hct) reference groups were set at 110–129 g/L (33%–39%) for the first or third trimesters and at 105–129 g/L (32%–39%) for the second trimester of pregnancy [4, 7, 19, 23]. For each analysis, women with missing Hb/Hct values were excluded. The modified Poisson regression models were also fitted to assess the relationships between weekly changes in Hb/Hct and stillbirth risk.

To explore a more flexible, non‐linear relationship between Hb/Hct and their changes and stillbirth risk, we fitted restricted cubic splines with three default knots (25th, 50th and 75th) using generalised linear models, with a Poisson distribution and a log‐link. Additionally, stillbirth risks by different Hb/Hct levels were also examined on the subgroups of singleton or multiple pregnancies, preterm or term stillbirths and SGA or non‐SGA stillbirths. Relative excess risk due to interaction (RERI) was calculated on the additive scale to assess Hb/Hct levels and plurality (singleton or multiple pregnancies). The RERI and 95% CI were based on 1000 bootstrap resampling. The sensitivity analyses were performed on 13 hospitals with test dates and on nulliparous women. Data analyses were conducted using R (version 4.4.3) and SPSS (version 27.0). DAG was developed using DAGitty.

### 2.5 Missing Data

Considering missing data in covariates, Hb/Hct values and outcomes, we performed sensitivity analysis using multiple imputation (MI), inverse probability weights (IPW) and inverse probability of censoring weights (IPCW) methods (details in Supporting Information).

### 2.6 Ethics Statement

The CBCS was approved by the Ethics Committee of Beijing Obstetrics and Gynaecology Hospital, Capital Medical University (reference 2018‐KY‐003‐02). All participants provided written informed consent to participate in this study and for the use of their clinical data.

## 3 Results

The final analyses included 142,715 women who had live births or stillbirths and at least one available Hb/Hct measurement during pregnancy (Figure S1). Overall, 23.6% of women experienced anaemia at least once during pregnancy, including mild (18.5%), moderate (5.0%) and severe (0.03%) anaemia (Table S1). The prevalence of maternal anaemia increased from 4.4% in the first trimester to 22.7% in the third trimester (Table S1). A higher anaemia rate was observed using Hct or combined Hb/Hct indicators (25.6% and 30.7%, respectively) (Tables S2 and S3).

Maternal socio‐demographic characteristics by foetal outcomes were shown (Table 1). Median weeks of gestation at Hb measurement were 10, 20 and 31 weeks for women with live births and 9, 18 and 30 weeks for those with stillbirths across the first, second and third trimesters, respectively (Table 1).

**TABLE 1:** Maternal characteristics of live births and stillbirths.

	Live birth (*n* = 142,098), *n* (%)	Stillbirth (*n* = 617), *n* (%)
Age, years		
< 35	121,714 (85.7)	487 (78.9)
≥ 35	20,358 (14.3)	130 (21.1)
Missing	26 (0.0)	0 (0.0)
BMI, kg/m^2^		
< 18.5	18,326 (12.9)	51 (8.3)
18.5–24.9	104,279 (73.4)	417 (67.6)
25.0–29.9	15,888 (11.2)	122 (19.8)
≥ 30.0	3581 (2.5)	27 (4.4)
Missing	24 (0.0)	0 (0.0)
Gravidity	1 (1–2)	2 (1–2)
Missing	894 (0.6)	7 (1.1)
Parity	0 (0–1)	0 (0–1)
Missing	948 (0.6)	7 (1.1)
Smoking	251 (0.2)	1 (0.2)
Missing	53 (0.0)	0 (0.0)
Drinking	5193 (3.7)	16 (2.6)
Missing	70 (0.0)	0 (0.0)
Region		
Eastern China	81,830 (57.6)	383 (62.1)
Central China	18,987 (13.4)	68 (11.0)
Western China	41,281 (29.1)	166 (26.9)
Ethnicity		
Han	134,877 (94.9)	588 (95.3)
Others	6619 (4.7)	28 (4.5)
Missing	602 (0.4)	1 (0.2)
Education		
Primary school or lower	331 (0.2)	4 (0.6)
High school or lower	19,817 (13.9)	95 (15.4)
College or above	121,909 (85.8)	518 (84.0)
Missing	41 (0.0)	0 (0.0)
Occupation		
Employed	113,412 (79.8)	478 (77.5)
Unemployed	28,667 (20.2)	139 (22.5)
Missing	19 (0.0)	0 (0.0)
Annual family income, CNY		
< 100,000	36,757 (25.9)	167 (27.1)
100,000–400,000	83,740 (58.9)	351 (56.9)
≥ 400,000	20,809 (14.6)	98 (15.9)
Missing	792 (0.6)	1 (0.2)
Mode of conception		
Natural pregnancy	133,373 (93.9)	533 (86.4)
ART	8677 (6.1)	84 (13.6)
Missing	48 (0.0)	0 (0.0)
Plurality		
Singleton	139,143 (97.9)	542 (87.8)
Multiple pregnancy	2952 (2.1)	73 (11.8)
Missing	3 (0)	2 (0.3)
Folic acid use	130,232 (92.2)	571 (92.5)
Missing	859 (0.6)	0 (0.0)
Multivitamin use	90,861 (64.9)	420 (68.3)
Missing	2018 (1.4)	2 (0.3)
Medication use in the 1st trimester	56,258 (39.9)	303 (49.7)
Missing	1011 (0.7)	7 (1.1)
Pre‐existing diseases	16,625 (11.7)	92 (14.9)
Missing	176 (0.1)	1 (0.2)
Gestational age at Hb measurement in the 1st trimester	10 (8–12)	9 (7–11)
Missing	35,394 (24.9)	123 (19.9)
Gestational age at Hb measurement in the 2nd trimester	20 (16–24)	18 (16–21)
Missing	47,278 (33.3)	179 (29.0)
Gestational age at Hb measurement in the 3rd trimester	31 (29–32)	30 (29–31)
Missing	32,598 (22.9)	486 (78.8)

*Note:* Data are median (IQR) or *n* (%). Percentages might add up to more than 100% due to rounding.

Abbreviations: 1st trimester, first trimester; 2nd trimester, second trimester; 3rd trimester, third trimester; ART, Assisted Reproductive Technology; BMI, Body Mass Index; CNY, Chinese Yuan; Hb, haemoglobin; IQR, Interquartile Range.

**FIGURE 1:**
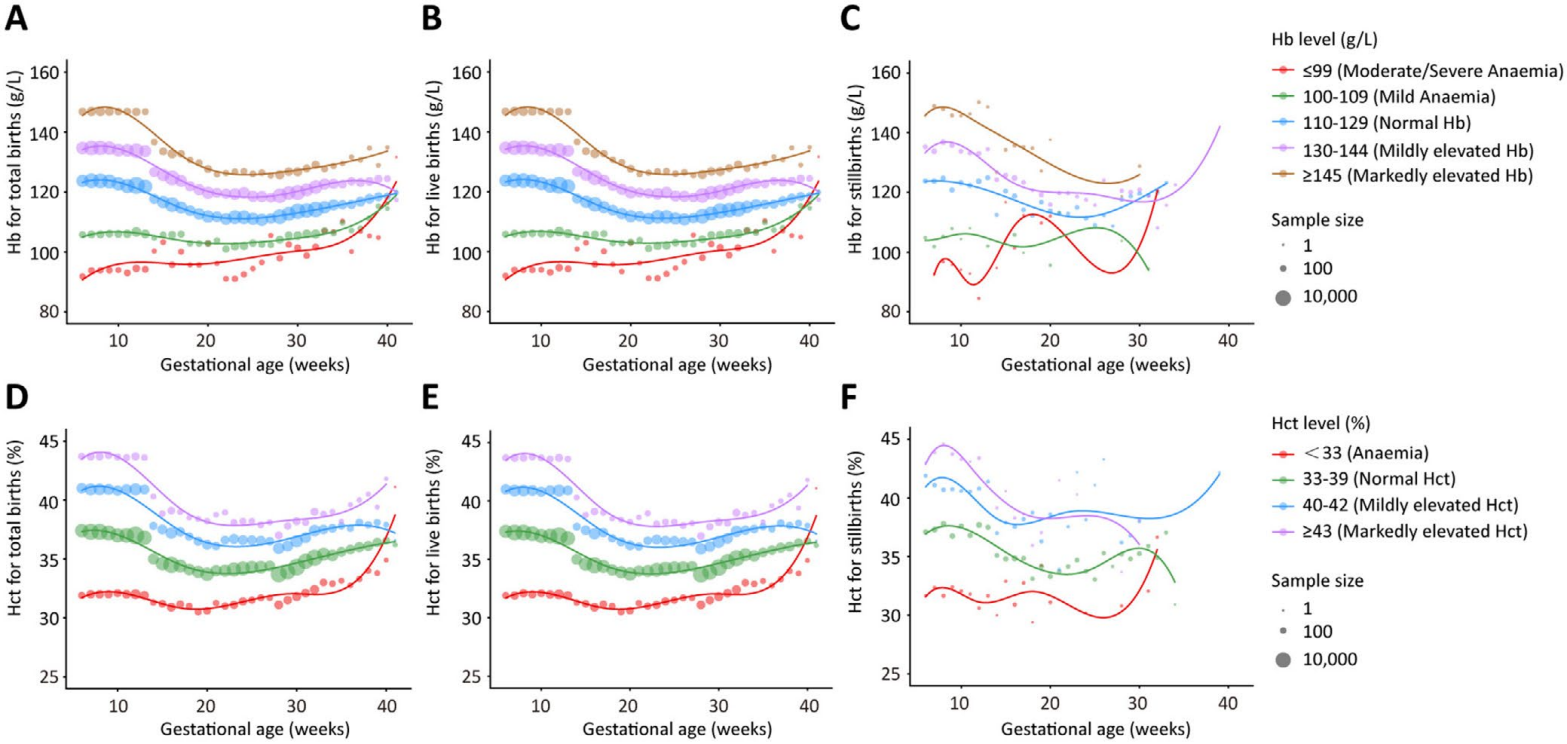
Haemoglobin concentration or hematocrit values during pregnancy. Hb concentration during pregnancy for total births, live births and stillbirths (A, B and C) and Hct values during pregnancy for total births, live births and stillbirths (D, E and F). Data were grouped by Hb concentration or Hct levels in the 1st trimester. Smooth splines were fitted to data grouped into one‐week intervals. The circle size represents participant number. Hb, haemoglobin; Hct, haematocrit; 1st trimester, first trimester.

The trajectories of Hb/Hct levels during pregnancy showed that maternal Hb values dropped from early pregnancy to late pregnancy, with Hct following a similar pattern (Figure 1). For groups of high or low levels at the extreme, Hb/Hct values tended to regress toward the mean (Figure 1). Women with live births and stillbirths had similar longitudinal distributions of Hb/Hct (Figure 1B,C, Figure 1E,F). Due to the small sample size of participants delivering stillbirths, greater fluctuation occurred in the moderate–severe anaemia and the highest Hct groups (Figure 1C and F).

**FIGURE 2:**
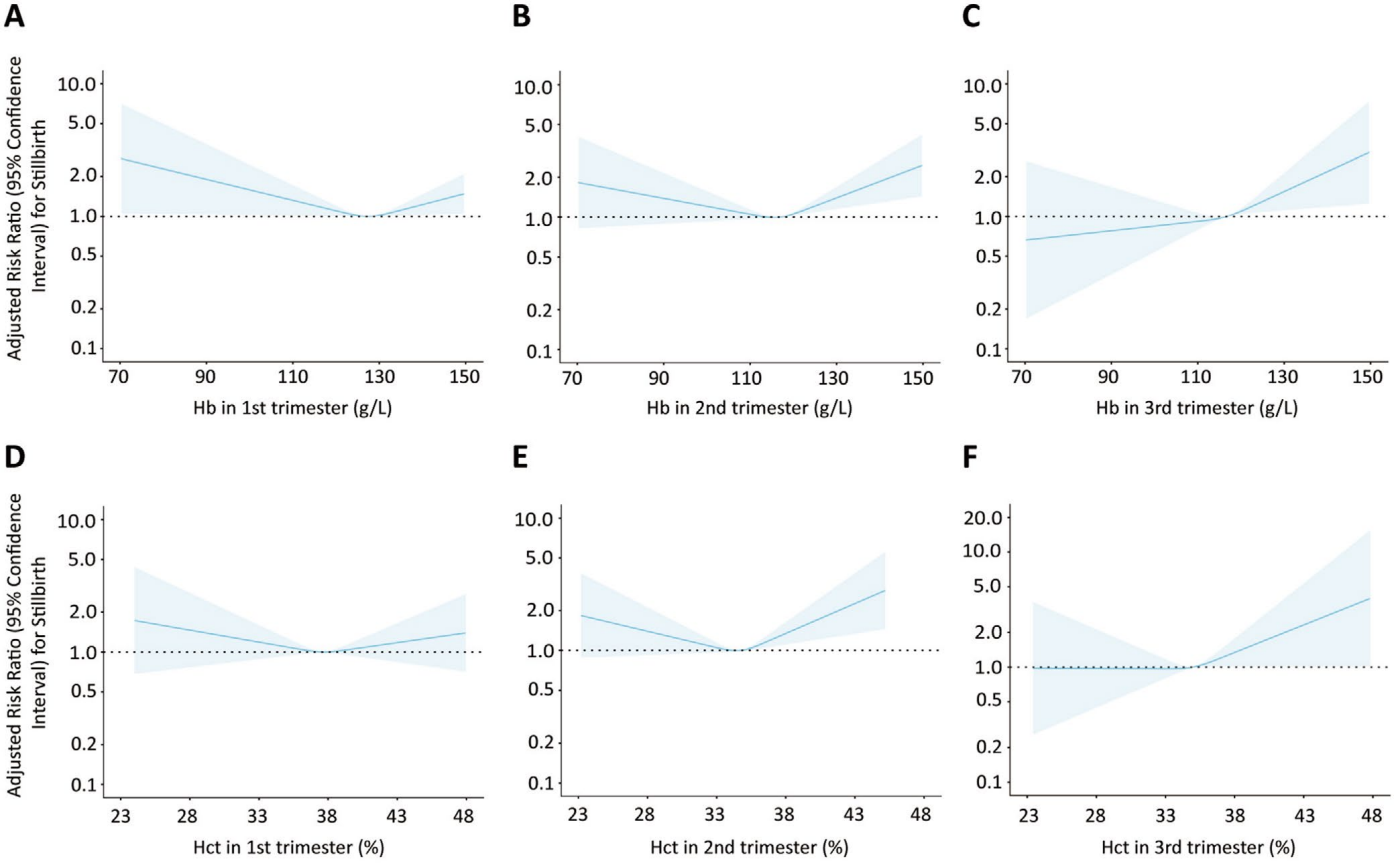
Association between Hb or Hct concentration in the 1st, 2nd and 3rd trimesters and stillbirth. Risk ratio (RR) with 95% CI for stillbirth by maternal Hb concentrations during 1st, 2nd and 3rd trimesters (A, B and C) and Hct levels during 1st, 2nd and 3rd trimesters (D, E and F). Associations were adjusted for maternal age, BMI, gravidity, drinking, region, ethnicity, annual family income, mode of conception, plurality, folic acid use, multivitamin use, medication use in the 1st trimester, pre‐existing diseases and gestational age at Hb/Hct measurement. The y‐axes are displayed on a logarithmic scale (base 10). Hb, haemoglobin; Hct, haematocrit; RR, risk ratio; CI, Confidence interval; BMI, Body Mass Index; 1st trimester, first trimester; 2nd trimester, second trimester; 3rd trimester, third trimester.

**FIGURE 3:**
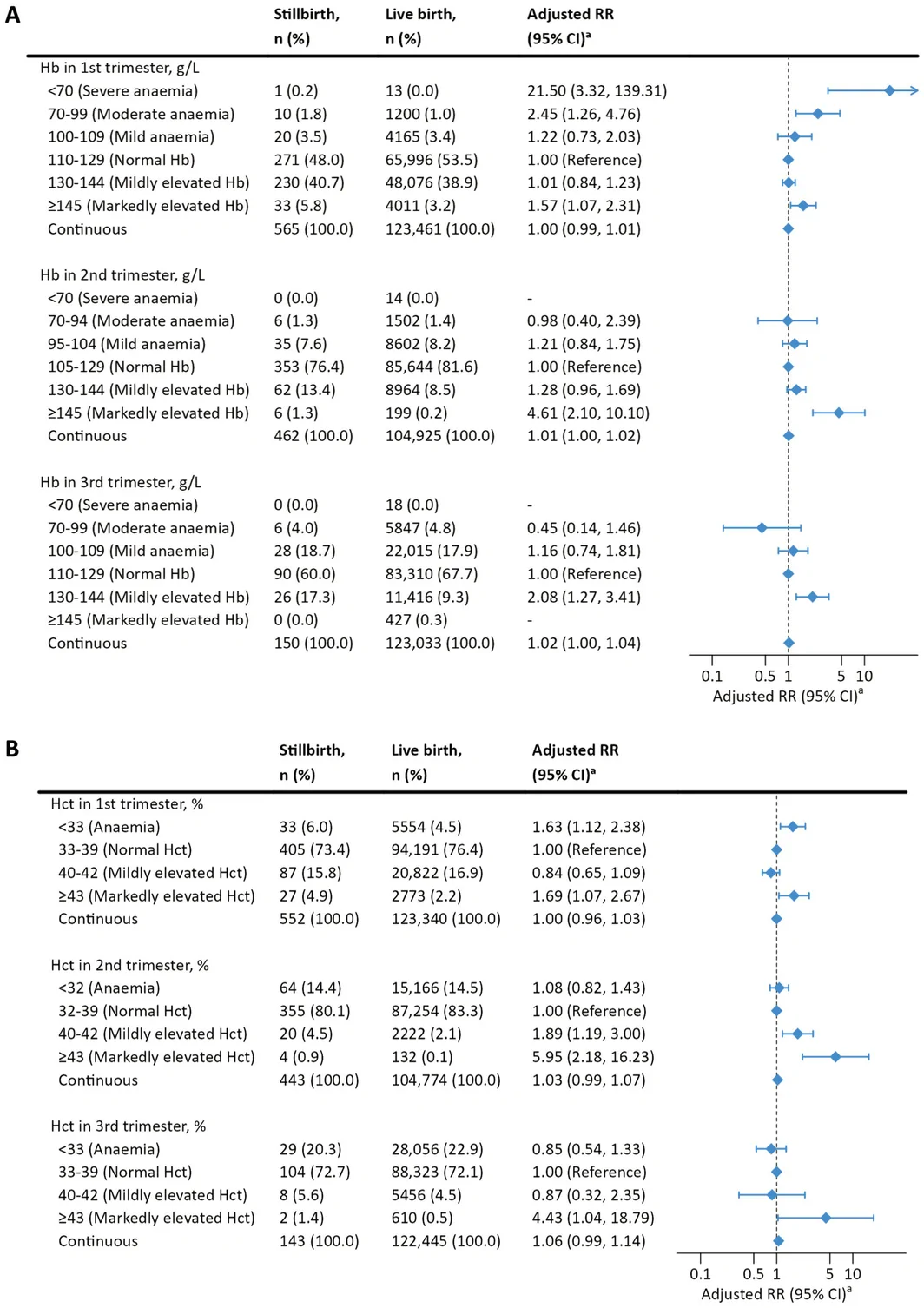
Stillbirth risk associated with Hb (A) or Hct (B) levels for various pregnancy trimesters. Data are *n* (%) or RR (95% CI). ^a^Associations were adjusted for maternal age, BMI, gravidity, drinking, region, ethnicity, annual family income, mode of conception, plurality, folic acid use, multivitamin use, medication use in 1st trimester, pre‐existing diseases, and gestational age at Hb/Hct measurement. The x‐axes are displayed on a logarithmic scale (base 10). Hb, haemoglobin; Hct, haematocrit; RR, risk ratio; CI, Confidence interval; BMI, body mass index; 1st trimester, first trimester; 2nd trimester, second trimester; 3rd trimester, third trimester.

A U‐shaped curve was observed, with increased stillbirth risk at both low and high Hb levels during the first and second trimesters (Figure 2). In the first trimester, the severity of anaemia and stillbirth risk followed a dose–effect relationship, while markedly high Hb was also associated with increased risk of stillbirth (Figure 3). No association between anaemia and stillbirth was found within the second and third trimesters (Figure 3). Compared with the reference group, the risk was more than fourfold for markedly elevated Hb in the second trimester and twofold for mildly elevated Hb in the third trimester (Figure 3).

There was a similar U‐shaped relationship between second‐trimester Hct and stillbirth (Figure 2). Maternal anaemia in the first trimester was associated with a higher stillbirth risk, and markedly elevated Hct in the first and third trimesters also increased the risk of stillbirth compared with the reference group (Figure 3). There was a gradual escalation in the stillbirth risk for pregnant women who had mildly elevated Hct and markedly elevated Hct in the second trimester (Figure 3).

**FIGURE 4:**
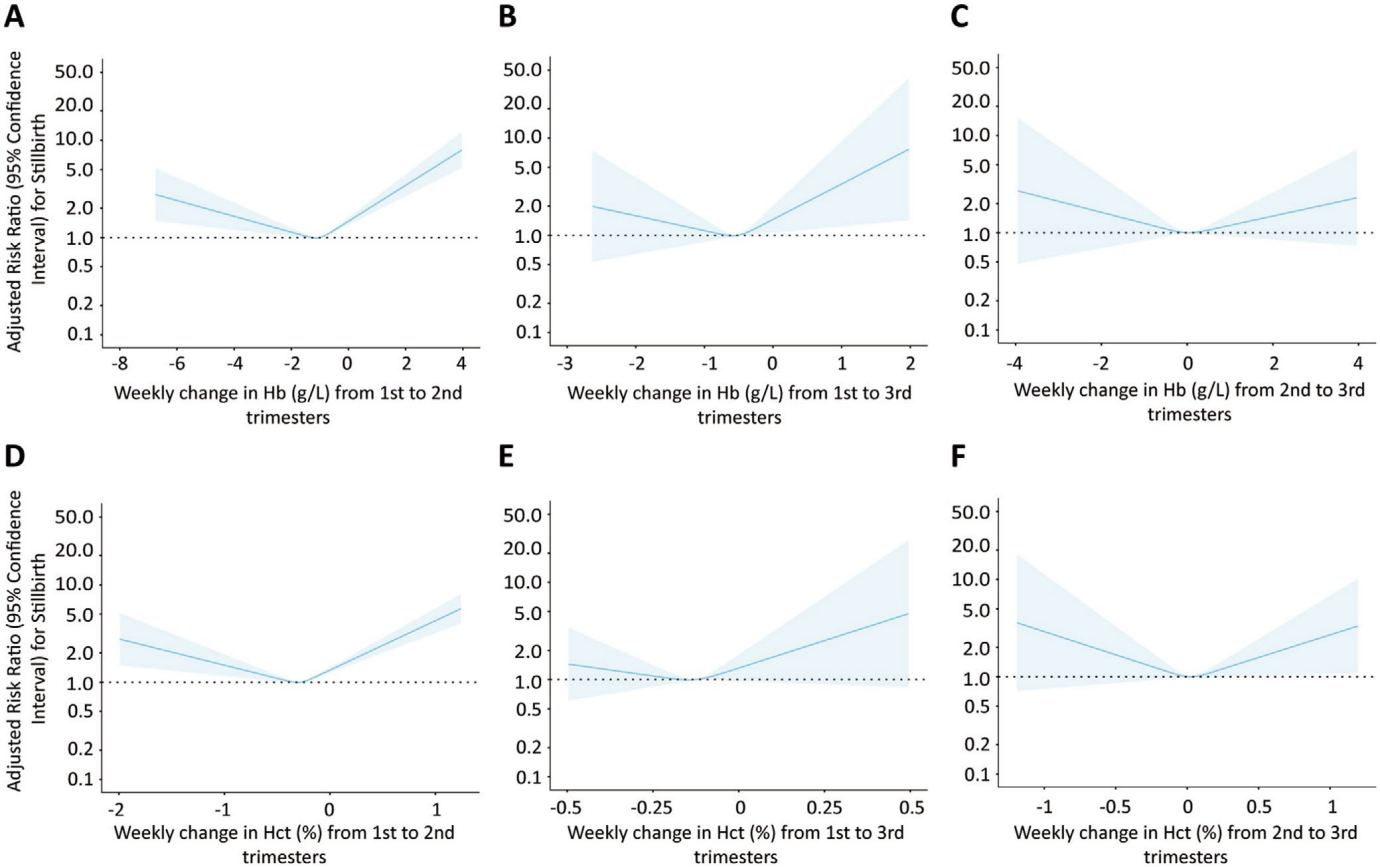
Association between Hb or Hct changes during pregnancy and stillbirth. Risk ratio (RR) with 95% CI for stillbirth by maternal Hb changes (2nd‐1st), (3rd‐1st) and (3rd‐2nd) trimesters (A, B, and C) and Hct changes (2nd‐1st), (3rd‐1st) and (3rd‐2nd) trimesters (D, E, and F). Associations were adjusted for maternal age, BMI, gravidity, drinking, region, ethnicity, annual family income, mode of conception, plurality, folic acid use, multivitamin use, medication use in 1st trimester, pre‐existing diseases, gestational age at first Hb/Hct measurement and Hb/Hct level in 1st trimester. The y‐axes are displayed on a logarithmic scale (base 10). Hb, haemoglobin; Hct, haematocrit; RR, risk ratio; CI, Confidence interval; BMI, Body Mass Index; 1st trimester, first trimester; 2nd trimester, second trimester; 3rd trimester, third trimester.

**FIGURE 5:**
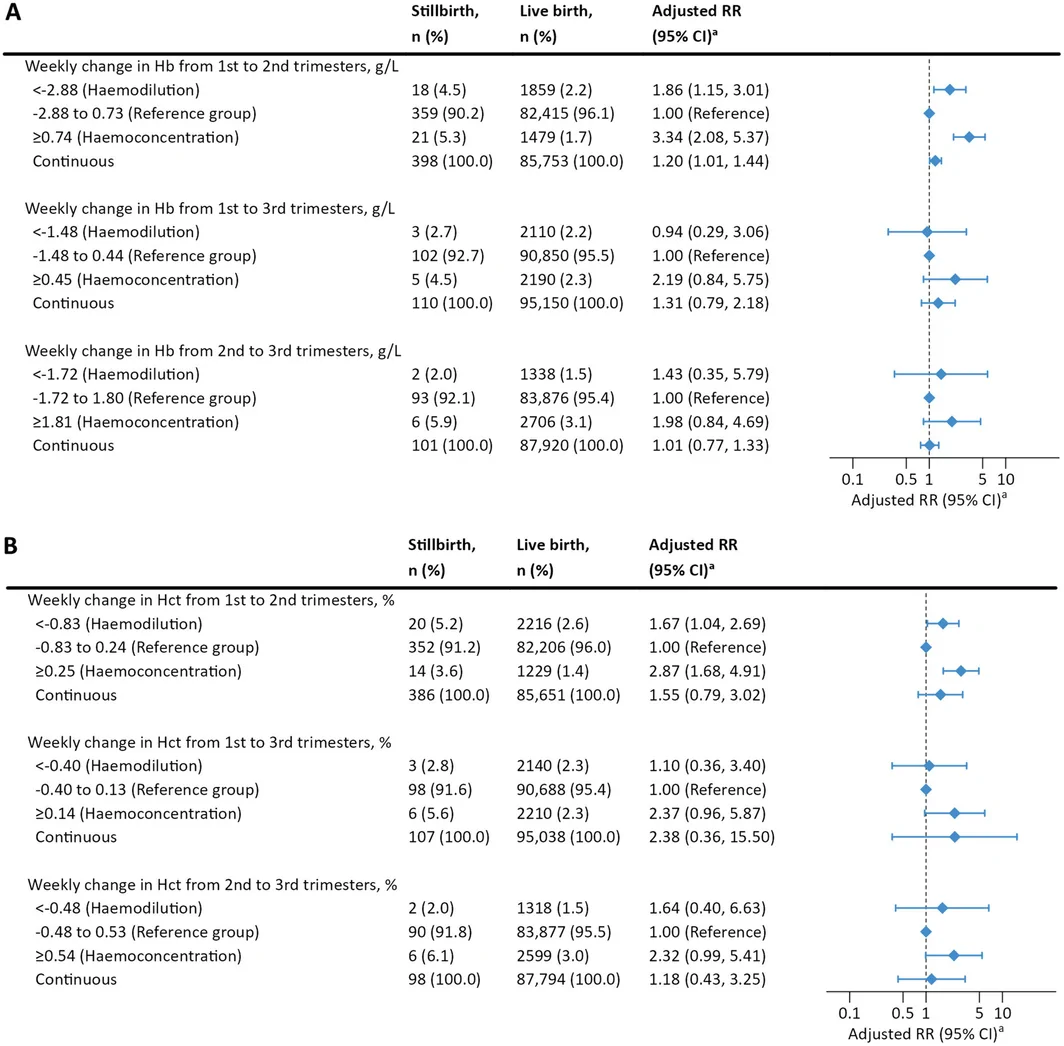
Association between weekly change in Hb (A) and Hct (B) levels during pregnancy and risk of stillbirth. Data are *n* (%) or RR (95% CI). ^a^Associations were adjusted for maternal age, BMI, gravidity, drinking, region, ethnicity, annual family income, mode of conception, plurality, folic acid use, multivitamin use, medication use in 1st trimester, pre‐existing diseases, gestational age at first Hb/Hct measurement and Hb/Hct level in the 1st trimester. The x‐axes are displayed on a logarithmic scale (base 10). Hb, haemoglobin; Hct, haematocrit; RR, risk ratio; CI, Confidence interval; BMI, Body Mass Index; 1st trimester, first trimester; 2nd trimester, second trimester; 3rd trimester, third trimester.

A U‐shaped relationship was observed between weekly changes in Hb/Hct and stillbirth risk, with both higher levels of haemodilution and haemoconcentration associated with increased stillbirth risk (Figure 4). Compared with women with a moderate change in Hb from the first to the second trimesters, stillbirth risk was 86% higher among women with Hb decreases of > 2.88 g/L per week and more than threefold higher for Hb increases of ≥ 0.74 g/L per week (Figure 5). Similarly, Hct decreases of > 0.83% per week and Hct increases of ≥ 0.25% per week from the first to the second trimesters were also linked to higher stillbirth risk (Figure 5). Each unit increase in weekly Hb change from the first to the second trimesters was associated with a 20% higher risk of stillbirth (Figure 5).

Baseline characteristics were similar between the excluded group without Hb/Hct values and the included participants (Table S4). Results were basically consistent using MI, IPW, and IPCW methods (Tables S5 and S6), with no evidence of effect modification by plurality (Table S7). Similar trends were observed after stratification by singleton or multiple pregnancies, gestational age, and birthweight centile (Tables S7 and S8) and when analyses were restricted to hospitals with test dates and to nulliparous women (Figures S3 and S4). Due to only 31% stillbirths occurring in the third trimester (Figure S5), the results for Hb/Hct levels and their changes during the third trimester were more representative of late or term stillbirths for this study.

## 4 Comment

### 4.1 Principal Findings

This study showed U‐shaped relationships between maternal Hb/Hct levels and stillbirth risk, with variation across trimesters. In the first trimester of pregnancy, moderate anaemia increases the risk of stillbirth. However, across three trimesters, higher Hb/Hct was related to increased risk of stillbirth. Excessive haemodilution or haemoconcentration from the first to the second trimesters increased the risk of stillbirth. These findings highlight the complex, trimester‐specific relationship between maternal blood parameters and stillbirth risk.

### 4.2 Strengths of the Study

The major strength was that serial Hb and Hct data across three trimesters were prospectively collected within the same population‐based cohort study, allowing us to assess dose–response relationships between maternal Hb/Hct levels throughout pregnancy and stillbirth risk. Furthermore, the relationship between Hb/Hct dynamics across trimesters and stillbirth risk was also examined, which highlighted the influence of maternal haemodilution status in late pregnancy on foetal outcomes. This study also included maternal Hct values, which were previously commonly disregarded. Both haematological parameters were classified based on lower trimester‐specific cutoffs recommended by the WHO and CDC, as well as higher cutoffs.

### 4.3 Limitations of the Data

Firstly, while adjusting for multivitamin supplementation, we lacked data on the dose/timing of iron for anaemic women. In China, women are recommended to routinely supplement with folic acid at 0.4–0.8 mg/d or take multivitamins before and after pregnancy [26]. The most widely used multivitamin is Elevit, containing 0.8 mg of folic acid, 60 mg of iron and 17 other micronutrients [28]. Information on multivitamin supplementation was available in CBCS. Iron tablets would be prescribed if iron deficiency anaemia were diagnosed [26]. However, data on iron supplementation were not collected in CBCS among women with anaemia. An updated 2024 Cochrane review found iron supplementation reduced maternal anaemia (average RR 0.30, 95% CI 0.20, 0.47; 14 trials, 13,543 women) and iron deficiency at term (average RR 0.51, 95% CI 0.38, 0.68; 8 trials, 2873 women) but showed uncertain benefits for pregnancy outcomes [29]. Secondly, we lacked data on the aetiology of maternal anaemia, elevated Hb/Hct and stillbirth, making it difficult to explore potential mechanisms underlying the U‐shaped relationship.

### 4.4 Interpretation

We found that maternal anaemia was still a moderately severe health issue in China based on the WHO criterion [30], with a prevalence of 23.6% (Table S1), similar to the WHO estimates (18.5%, 95% CI 9.7,31.7) in 2019, and 21.66% estimated by a Chinese population‐based retrospective cohort study in 2019 [3, 5]. The anaemia rates were higher with Hct and both Hb/Hct indicators (25.6% and 30.7%, respectively), primarily due to the disproportionate change in plasma volume relative to red cell mass [22]. Lower Hct classified more women as anaemic due to haemodilution alone, even with normal oxygen‐carrying capacity and adequate iron stores. In China, prenatal clinical guidelines do not recommend routine iron supplementation unless iron‐deficiency anaemia is diagnosed [26]. Although multivitamin supplements were encouraged, our results suggested that only 64.9% of pregnant women opted to take them, which partially explained the high prevalence of anaemia during pregnancy.

The U‐shaped curves for Hb/Hct and stillbirth risk were consistent with previous studies [4, 6, 11]. Similar findings have been reported by various studies [5, 6, 11, 13], in which moderate–severe anaemia increased stillbirth risk [4, 5, 6, 11, 13, 24], while mild anaemia showed no effect [6, 13] or a reduced stillbirth risk [5, 11]. In 2019, a systematic review and meta‐analysis found that increased stillbirth risk for pregnant women was at low or high levels of Hb (adjusted odds ratio [aOR] for < 110 g/L: 1.49, 95% CI 1.15, 1.92; 21 studies; aOR for ≥ 130 g/L: 1.88, 95% CI 1.21, 2.91; 4 studies) [2]. The timing of Hb assessment varied, potentially leading to inconsistent results for anaemia severity and stillbirth risk. In our analysis, the risk of stillbirth was 69% higher for Hct > 43% in the first trimester and more than fivefold for Hct > 43% in the second trimester.

A study involving Iranian pregnant women observed that haemodilution during pregnancy was a protective factor for stillbirth, and yet the haemoconcentration group was set as reference, which might obscure the relative risks [14]. Another study reported that Swedish pregnant women with the largest Hb rises during early pregnancy (≥ 0.5 g/L) had a twofold higher risk of stillbirth, but no associations were detected after adjusting for confounders [12]. Unlike previous studies, we used cutoffs of more than 2 SD below (or above) the mean of Hb/Hct changes, with the middle group as the reference. Both excessive haemodilution and excessive haemoconcentration from the first to the second trimesters were identified as risk factors for stillbirth. We also analysed the RRs for stillbirth risk and the change in Hb/Hct across various trimesters as continuous variables, revealing a similar U‐shaped relationship.

So far, the biological mechanism underlying the U‐shaped relationship between Hb/Hct and their dynamic changes and the risk of stillbirth is not well understood. Physiological haemodilution during pregnancy occurs through plasma expansion, reduced blood viscosity and increased uteroplacental blood flow [4, 5, 12, 14]. This process involves compensatory changes to enhance foetal oxygen supply and uteroplacental perfusion [5, 12]. However, moderate–severe anaemia or excess haemodilution can exceed compensatory capacity, leading to foetal chronic hypoxia, impaired foetal development or stillbirth [24]. High Hb, often associated with preeclampsia and eclampsia, resulted from reduced blood volume and haemoconcentration [12, 13]. These conditions were known risk factors for stillbirth, so we did not adjust for them in the multivariable models [12]. Elevated Hb also partly stemmed from maternal iron overload, causing oxidative stress and placental cell DNA damage [1]. Nevertheless, further research is required to explore potential mechanisms linking Hb/Hct levels at various gestational ages, their changes across all trimesters and stillbirth risk.

Our study depicted longitudinal distributions of Hb/Hct across various gestational ages, emphasising the clinical relevance of monitoring dynamic changes in maternal Hb/Hct across the three trimesters. Early anaemia elevated stillbirth risk, necessitating proactive prevention/treatment. Elevated Hb/Hct also increased stillbirth likelihood, highlighting the need for standardised high‐value thresholds and targeted monitoring. Improved metrics for assessing haemodilution (plasma expansion) could enhance understanding of blood parameter fluctuations and adverse outcomes. Prioritising trimester‐specific guidelines and refining diagnostic tools may optimise maternal care and reduce preventable stillbirths linked to both anaemia and excessive Hb/Hct levels.

### 4.5 Conclusions

This large cohort study found that maternal anaemia was still a contemporary public health issue in China. Both lower and higher Hb/Hct increased stillbirth risk, though the association varied by trimester. Excess haemodilution and haemoconcentration in late pregnancy were risk factors for stillbirth.

## Author Contributions

S.L., N.D., R.L., A.E.P.H. and C.Y. developed the study conception and design. C.Y. conceived the CBCS Project and obtained funding. S.G., S.S., S.X., Z.Y., M.H., J.L., Z.L., E.Z., J.L., H.X. and J.Y. collected data. S.S., S.X., E.Z. and J.L. were involved in data curation. S.L., S.G. and S.S. were involved in statistical analysis. S.L. wrote the first draft of the manuscript. N.D., S.G., S.W., W.Y., R.L., A.E.P.H. and C.Y. provided critical revisions. S.L., S.S., S.X., E.Z., J.L., R.L. and C.Y. had directly accessed and verified the underlying data. All authors read and approved the submission of the manuscript.

## Funding

This work was supported by the National Key Research and Development Program of China (Grant 2016YFC1000101), China Scholarship Council (Grant 202408110195).

## Conflicts of Interest

A.E.P.H. is supported by Tommy's Centre funding for the Maternal and Fetal Health Research Centre, University of Manchester, research for Patient Benefit Funding—MiNESS20‐28 from the National Institute for Health Research, and funding for the national evaluation of Saving Babies Lives Care Bundle from NHS England. The remaining authors declare no conflicts of interest.

## Supporting information


**Figure S1:** Flowchart of study participants.
**Figure S2:** DAG for maternal Hb/Hct levels and their changes during pregnancy and stillbirth analyses.
**Figure S3:** Stillbirth risk associated with Hb (A) or Hct (B) levels for various trimesters in 13 hospitals.
**Figure S4:** Stillbirth risk associated with Hb (A) or Hct (B) for various trimesters in nulliparous women.
**Figure S5:** Distribution of gestational weeks at delivery for stillbirths and live births.
**Table S1:** Rate of anaemia during pregnancy based on maternal Hb concentrations.
**Table S2:** Rate of anaemia during pregnancy based on maternal Hct levels.
**Table S3:** Rate of anaemia during pregnancy based on maternal Hb and Hct levels.
**Table S4:** Maternal characteristics of the excluded group and included group.
**Table S5:** Stillbirth risk associated with Hb or Hct levels in three trimesters across different statistical models.
**Table S6:** Association between weekly change in Hb and Hct levels during pregnancy and risk of stillbirth across different statistical models.
**Table S7:** Subgroup analysis of stillbirth risk associated with Hb/Hct levels for singleton and multiple pregnancies.
**Table S8:** Subgroup analysis of stillbirth risk associated with Hb/Hct levels for various trimesters.

## Data Availability

The study data cannot be shared publicly due to reasons of sensitivity and to protect the participants' privacy. Data are located in a secure cloud‐based server with restricted access. The data can be available upon reasonable request and approval of the ethics committee and data custodians.
