# Supplementary material for: Maternal Haemoglobin and Haematocrit During Pregnancy and Stillbirth Risk: A Prospective, Population‐Based Birth Cohort Study
Source: Paediatr Perinat Epidemiol. 2026 Mar 31;40(6):709–19. doi: 10.1111/ppe.70144 (PMC13522651; doi:10.1111/ppe.70144)
Supplement: Supplementary file 1 [file PPE-40-709-s001.docx]

**Supplemental Methods**

**2.5 Missing Data**

For primary analysis, the missing data for the sociodemographic and clinical covariates were treated as a separate level in categorical variables. In the sensitivity analysis, we employed multiple imputation (MI) methods with 50 imputed data sets generated to address missing covariate data. Results from each imputed dataset were pooled based on Rubin’s Rules.

Considering a large proportion of women (n= 56,163) without one Hb/Hct value available were excluded, we used the standardized mean difference (SMD) to compare characteristics between included group and excluded group. We also performed a combination of MI and the stabilised inverse probability weights (IPW) to up-weight pregnant women who remained to represent similar women who were excluded as there were no Hb or Hct values. The weights included the same covariables as the Poisson models except Hb or Hct values and truncated at the 1st and 99th percentiles in each imputed data set to avoid extreme weights. To account for loss to follow-up (n=31,982), we adopted a combined approach of MI with 50 imputations and the stabilised inverse probability of censoring weights (IPCW). The censoring weights were assessed in each imputed data set, conditional on the same covariables as the modified Poisson regression models, except maternal Hb/Hct values in three trimesters, which were not available for women lost to follow-up. IPCW were truncated at the 1st and 99th percentiles.

**Supplemental Figures**

**eFigure 1.** Flowchart of study participants.

***Legend:*** ^a^142,030 women had both one Hb value and one Hct value available for the same measurement. A total of 685 women had only one Hb value without the corresponding Hct value. Abbreviations: Hb, haemoglobin; Hct, haematocrit.

**eFigure 2.** DAG for maternal Hb/Hct levels and their changes during pregnancy and stillbirth analyses.

***Legend:*** The minimally sufficient adjustment set included maternal age, BMI, gravidity, drinking, region, ethnicity, annual family income, mode of conception, plurality, folic acid use, multivitamin use, medication use in 1st trimester, pre-existing diseases, and gestational age at Hb/Hct measurement. Abbreviations: Hb, haemoglobin; Hct, haematocrit; 1st trimester, first trimester; 2nd trimester, second trimester; 3rd trimester, third trimester; U, unmeasured confounders; DAG, Directed Acyclic Graph.

**eFigure 3.** Stillbirth risk associated with Hb (panel A) or Hct (panel B) levels for various trimesters in 13 hospitals.

***Legend:*** Data are n (%) or RR (95% CI). All test dates of Hb/Hct were retrieved for pregnant women in 13 hospitals and only the test trimesters were available for the others. ^a^Associations were adjusted for maternal age, BMI, gravidity, drinking, region, ethnicity, annual family income, mode of conception, plurality, folic acid use, multivitamin use, medication use in 1st trimester, pre-existing diseases, and gestational age at Hb/Hct measurement. The x-axes are displayed on a logarithmic scale (base 10). Abbreviations: RR, risk ratio; CI: confidence interval; BMI, Body Mass Index; 1st trimester, first trimester; 2nd trimester, second trimester; 3rd trimester, third trimester.

**eFigure 4.** Stillbirth risk associated with Hb (panel A) or Hct (panel B) for various trimesters in nulliparous women.

***Legend:*** Data are n (%) or RR (95% CI). ^a^Associations were adjusted for maternal age, BMI, gravidity, drinking, region, ethnicity, annual family income, mode of conception, plurality, folic acid use, multivitamin use, medication use in 1st trimester, pre-existing diseases, and gestational age at Hb/Hct measurement. The x-axes are displayed on a logarithmic scale (base 10). Abbreviations: RR, risk ratio; CI: confidence interval; BMI, Body Mass Index; 1st trimester, first trimester; 2nd trimester, second trimester; 3rd trimester, third trimester.

**eFigure 5.** Distribution of gestational weeks at delivery for stillbirths and live births.

**Supplemental Tables**

**eTable 1.** Rate of anaemia during pregnancy based on maternal Hb concentrations.

|  | **Patients, n (%)** |  |  |  |
| --- | --- | --- | --- | --- |
|  | **During pregnancy** | **1st trimester** | **2nd trimester** | **3rd trimester** |
| Anaemia | 33,680 (23.6) | 5409 (4.4) | 10,159 (9.6) | 27,914 (22.7) |
| Mild anaemia | 26,472 (18.5) | 4185 (3.4) | 8637 (8.2) | 22,043 (17.9) |
| Moderate anaemia | 7167 (5.0) | 1210 (1.0) | 1508 (1.4) | 5853 (4.8) |
| Severe anaemia | 41 (0.0) | 14 (0.0) | 14 (0.0) | 18 (0.0) |
| No anaemia | 109,035 (76.4) | 11,8617 (95.6) | 95,228 (90.4) | 95,269 (77.3) |
| Total | 142,715 (100.0) | 124,026 (100.0) | 105,387 (100.0) | 123,183 (100.0) |

Data are n (%). Percentages might add up to more than 100% due to rounding. Anaemia was defined as a Hb concentration of less than 110 g/L in 1st or 3rd trimester (or less than 105 g/L in 2nd trimester). Mild anaemia indicates a Hb of 100 to 109 g/L in 1st or 3rd trimester (or 95 to 104 g/L in 2nd trimester). Moderate anaemia indicates a Hb of 70 to 99 g/L in 1st or 3rd trimester (or 70 to 94 g/L in 2nd trimester). Severe anaemia indicates a Hb of less than 70 g/L. No anaemia was defined as a Hb concentration of 110 g/L or greater in 1st or 3rd trimester (or 105 g/L or greater in 2nd trimester). Abbreviations: Hb, haemoglobin; 1st trimester, first trimester; 2nd trimester, second trimester; 3rd trimester, third trimester.

**eTable 2.** Rate of anaemia during pregnancy based on maternal Hct levels.

|  | **Patients, n (%)** |  |  |  |
| --- | --- | --- | --- | --- |
|  | **During pregnancy** | **1st trimester** | **2nd trimester** | **3rd trimester** |
| Anaemia | 36,359 (25.6) | 5587 (4.5) | 15,230 (14.5) | 28,085 (22.9) |
| No anaemia | 105,671 (74.4) | 118,305 (95.6) | 89,987 (85.5) | 94,503 (77.1) |
| Total | 142,030 (100.0) | 123,892 (100.0) | 105,217 (100.0) | 122,588 (100.0) |

Data are n (%). Percentages might add up to more than 100% due to rounding. Anaemia indicates a Hct level of less than 33% in 1st or 3rd trimester (or less than 32% in 2nd trimester). No anaemia was defined as a Hct level of 33% or greater in 1st or 3rd trimester (or 32% or greater in 2nd trimester). Abbreviations: Hct, haematocrit; 1st trimester, first trimester; 2nd trimester, second trimester; 3rd trimester, third trimester.

**eTable 3.** Rate of anaemia during pregnancy based on maternal Hb and Hct levels.

|  | **Patients, n (%)** |  |  |  |
| --- | --- | --- | --- | --- |
|  | **During pregnancy** | **1st trimester** | **2nd trimester** | **3rd trimester** |
| Anaemia | 43,872 (30.7) | 7421 (6.0) | 17,185 (16.3) | 35,051 (28.5) |
| Mild anaemia | 26,472 (18.5) | 4185 (3.4) | 8637 (8.2) | 22,043 (17.9) |
| Moderate anaemia | 7167 (5.0) | 1210 (1.0) | 1508 (1.4) | 5853 (4.8) |
| Severe anaemia | 41 (0.0) | 14 (0.0) | 14 (0.0) | 18 (0.0) |
| anaemia of unknown severity | 10,192 (7.1) | 2012 (1.6) | 7026 (6.7) | 7137 (5.8) |
| No anaemia | 98,843 (69.3) | 116,605 (94.0) | 88,202 (83.7) | 88,132 (71.5) |
| Total | 142,715 (100.0) | 124,026 (100.0) | 105,387 (100.0) | 123,183 (100.0) |

Data are n (%). Percentages might add up to more than 100% due to rounding. Anaemia was defined as a Hb concentration of less than 110 g/L in 1st or 3rd trimester, or less than 105 g/L in 2nd trimester (or a Hct level of less than 33% in 1st or 3rd trimester, or less than 32% in 2nd trimester). Mild anaemia indicates a Hb of 100 to 109 g/L in 1st or 3rd trimester (or 95 to 104 g/L in 2nd trimester). Moderate anaemia indicates a Hb of 70 to 99 g/L in 1st or 3rd trimester (or 70 to 94 g/L in 2nd trimester). Severe anaemia indicates a Hb of less than 70 g/L. No anaemia was defined as a Hb concentration of 110 g/L or greater in 1st or 3rd trimester, or 105 g/L or greater in 2nd trimester (a Hct level of 33% or greater in 1st or 3rd trimester or 32% or greater in 2nd trimester). Abbreviations: Hb, haemoglobin; Hct, haematocrit; 1st trimester, first trimester; 2nd trimester, second trimester; 3rd trimester, third trimester.

**eTable 4.** Maternal characteristics of excluded group and included group.

|  | **Excluded group without Hb/Hct values (n=56,163), n (%)** | **Included group with Hb/Hct values (n=142,715),**  **n (%)** | **SMD** |
| --- | --- | --- | --- |
| Age, years |  |  |  |
| <35 | 49,102 (87.4) | 122,201 (85.6) | 0.054 |
| ≥35 | 7044 (12.5) | 20,488 (14.4) |  |
| Missing | 17 (0.0) | 26 (0.0) |  |
| BMI, kg/m^2^ |  |  |  |
| < 18.5 | 7309 (13.0) | 18,377 (12.9) | 0.032 |
| 18.5-24.9 | 40,679 (72.4) | 104,696 (73.4) |  |
| 25.0-29.9 | 6,829 (12.2) | 16,010 (11.2) |  |
| ≥30.0 | 1,341 (2.4) | 3608 (2.5) |  |
| Missing | 5 (0.0) | 24 (0.0) |  |
| Gravidity | 2 (1-2) | 1 (1-2) | 0.129 |
| Missing | 34(0.1) | 38(0.1) |  |
| Parity | 0 (0-1) | 0 (0-1) | 0.173 |
| Missing | 901(0.6) | 955(0.7) |  |
| Smoking | 125 (0.2) | 252 (0.2) | 0.019 |
| Missing | 43 (0.1) | 53 (0.0) |  |
| Drinking | 1595 (2.8) | 5209 (3.6) | 0.047 |
| Missing | 42 (0.1) | 70 (0.0) |  |
| Region |  |  | 0.284 |
| Eastern China | 24,465 (43.6) | 82,213 (57.6) |  |
| Central China | 9586 (17.1) | 19,055 (13.4) |  |
| Western China | 22,112 (39.4) | 41,447 (29.0) |  |
| Ethnicity |  |  | 0.174 |
| Han | 50,780 (90.4) | 135,465 (94.9) |  |
| Others | 4998 (8.9) | 6647 (4.7) |  |
| Missing | 385 (0.7) | 603 (0.4) |  |
| Education |  |  | 0.247 |
| Primary school or lower | 399 (0.7) | 335 (0.2) |  |
| High school or lower | 12,887 (22.9) | 19,912 (14.0) |  |
| College or above | 42,875 (76.3) | 122,427 (85.8) |  |
| Missing | 2 (0.0) | 41 (0.0) |  |
| Occupation |  |  | 0.227 |
| Employed | 39,328 (70.0) | 113,890 (79.8) |  |
| Unemployed | 16,830 (30.0) | 28,806 (20.2) |  |
| Missing | 5 (0.0) | 19 (0.0) |  |
| Annual family income, CNY |  |  | 0.342 |
| <100,000 | 23,268 (41.4) | 36,924 (25.9) |  |
| 100,000-400,000 | 26,400 (47.0) | 84,091 (58.9) |  |
| ≥400,000 | 5961 (10.6) | 20,907 (14.6) |  |
| Missing | 534 (1.0) | 793 (0.6) |  |
| Mode of conception |  |  | 0.081 |
| Natural pregnancy | 53,692 (95.6) | 133,906 (93.8) |  |
| ART | 2468 (4.4) | 8761 (6.1) |  |
| Missing | 3 (0.0) | 48 (0.0) |  |
| Plurality |  |  | 0.035 |
| Singleton | 55,235 (98.3) | 139,685 (97.9) |  |
| Multiple pregnancy | 928 (1.7) | 3025 (2.1) |  |
| Missing | 0 (0.0) | 5 (0.0) |  |
| Folic acid use | 4762 (8.5) | 11,053 (7.7) | 0.054 |
| Missing | 573 (1.0) | 859 (0.6) |  |
| Multivitamin use | 22,866 (40.7) | 49,414 (34.6) | 0.148 |
| Missing | 1279 (2.3) | 2020 (1.4) |  |
| Medication use in 1st trimester | 22,281 (39.7) | 56,561 (39.6) | 0.020 |
| Missing | 312 (0.6) | 1018 (0.7) |  |
| Pre-existing diseases | 5710 (10.2) | 16,717 (11.7) | 0.054 |
| Missing | 35 (0.1) | 177 (0.1) |  |

Data are median (IQR) or n (%). Percentages might add up to more than 100% due to rounding. Abbreviations: SMD, standardised mean difference; BMI, Body Mass Index; ART, Assisted Reproductive Technology; 1st trimester, first trimester; IQR, Interquartile Range.

**eTable 5.** Stillbirth risk associated with Hb or Hct levels in three trimesters across different statistical models.

|  | **Adjusted RR (95%CI)** | | |
| --- | --- | --- | --- |
|  | **Model1^a^** | **Model2^b^** | **Model3^c^** |
| Hb in 1st trimester, g/L |  |  |  |
| <70 (Severe anaemia) | 18.79 (2.87, 123.16) | 22.52 (3.60, 140.94) | 20.86 (3.27, 133.03) |
| 70-99 (Moderate anaemia) | 2.04 (1.08, 3.83) | 2.11 (1.11, 4.00) | 1.99 (1.05, 3.78) |
| 100-109 (Mild anaemia) | 1.20 (0.76, 1.89) | 1.15 (0.72, 1.83) | 1.17 (0.74, 1.87) |
| 110-129 (Normal Hb) | 1.00 (Reference) | 1.00 (Reference) | 1.00 (Reference) |
| 130-144 (Mildly elevated Hb) | 1.02 (0.85, 1.22) | 1.00 (0.84, 1.20) | 1.01 (0.84, 1.21) |
| ≥145 (Markedly elevated Hb) | 1.47 (1.02, 2.13) | 1.53 (1.05, 2.22) | 1.52 (1.05, 2.19) |
| Continuous | 1.00 (0.99, 1.01) | 1.00 (0.99, 1.01) | 1.00 (0.99, 1.01) |
| Hb in 2nd trimester, g/L |  |  |  |
| <70 (Severe anaemia) | - | - | - |
| 70-94 (Moderate anaemia) | 1.06 (0.47, 2.41) | 1.19 (0.52, 2.77) | 1.13 (0.48, 2.64) |
| 95-104 (Mild anaemia) | 1.14 (0.79, 1.63) | 1.06 (0.73, 1.54) | 1.06 (0.74, 1.53) |
| 105-129 (Normal Hb) | 1.00 (Reference) | 1.00 (Reference) | 1.00 (Reference) |
| 130-144 (Mildly elevated Hb) | 1.31 (0.99, 1.73) | 1.36 (1.03, 1.81) | 1.35 (1.02, 1.79) |
| ≥145 (Markedly elevated Hb) | 4.57 (2.09, 10.01) | 4.21 (1.91, 9.28) | 4.35 (1.98, 9.58) |
| Continuous | 1.01 (1.00, 1.02) | 1.01 (1.00, 1.02) | 1.01 (1.00, 1.02) |
| Hb in 3rd trimester, g/L |  |  |  |
| <70 (Severe anaemia) | - | - | - |
| 70-99 (Moderate anaemia) | 0.83 (0.36, 1.94) | 0.95 (0.40, 2.27) | 0.90 (0.38, 2.11) |
| 100-109 (Mild anaemia) | 1.12 (0.73, 1.71) | 1.09 (0.70, 1.67) | 1.09 (0.71, 1.68) |
| 110-129 (Normal Hb) | 1.00 (Reference) | 1.00 (Reference) | 1.00 (Reference) |
| 130-144 (Mildly elevated Hb) | 2.35 (1.51, 3.64) | 2.32 (1.49, 3.62) | 2.28 (1.47, 3.55) |
| ≥145 (Markedly elevated Hb) | - | - | - |
| Continuous | 1.02 (1.01, 1.04) | 1.02 (1.00, 1.04) | 1.02 (1.00, 1.04) |
| Hct in 1st trimester, % |  |  |  |
| <33 (Anaemia) | 1.45 (1.01, 2.07) | 1.40 (0.98, 2.02) | 1.38 (0.96, 1.99) |
| 33-39 (Normal Hct) | 1.00 (Reference) | 1.00 (Reference) | 1.00 (Reference) |
| 40-42 (Mildly elevated Hct) | 0.87 (0.69, 1.10) | 0.87 (0.69, 1.11) | 0.87 (0.69, 1.10) |
| ≥43 (Markedly elevated Hct) | 1.82 (1.23, 2.69) | 1.90 (1.27, 2.82) | 1.90 (1.28, 2.81) |
| Continuous | 1.00 (0.97, 1.04) | 1.00 (0.97, 1.04) | 1.00 (0.97, 1.04) |
| Hct in 2nd trimester, % |  |  |  |
| <32 (Anaemia) | 1.03 (0.79, 1.35) | 1.04 (0.79, 1.37) | 1.03 (0.79, 1.36) |
| 32-39 (Normal Hct) | 1.00 (Reference) | 1.00 (Reference) | 1.00 (Reference) |
| 40-42 (Mildly elevated Hct) | 1.87 (1.19, 2.95) | 1.87 (1.17, 2.98) | 1.92 (1.20, 3.08) |
| ≥43 (Markedly elevated Hct) | 5.33 (1.96, 14.49) | 5.20 (1.89, 14.34) | 5.22 (1.90, 14.32) |
| Continuous | 1.04 (1.00, 1.08) | 1.04 (1.00, 1.08) | 1.04 (1.00, 1.08) |
| Hct in 3rd trimester, % |  |  |  |
| <33 (Anaemia) | 0.80 (0.53, 1.23) | 0.79 (0.51, 1.20) | 0.80 (0.53, 1.23) |
| 33-39 (Normal Hct) | 1.00 (Reference) | 1.00 (Reference) | 1.00 (Reference) |
| 40-42 (Mildly elevated Hct) | 1.45 (0.71, 2.96) | 1.51 (0.73, 3.11) | 1.46 (0.71, 3.00) |
| ≥43 (Markedly elevated Hct) | 3.61 (0.87, 15.07) | 3.69 (0.89, 15.29) | 3.23 (0.78, 13.42) |
| Continuous | 1.09 (1.02, 1.16) | 1.09 (1.02, 1.16) | 1.08 (1.01, 1.15) |

Data are n (%) or RR (95% CI). All models were adjusted for maternal age, BMI, gravidity, drinking, region, ethnicity, annual family income, mode of conception, plurality, folic acid use, multivitamin use, medication use in 1st trimester, pre-existing diseases, and gestational age at Hb/Hct measurement. ^a^Relative risk pooled from 50 imputed data sets. ^b^Relative risk pooled from 50 imputed data sets, weighted by IPW to account for exclude group without Hb/Hct values. ^c^Relative risk pooled from 50 imputed data sets, weighted by IPCW to account for loss to follow-up. Abbreviations: Hb, haemoglobin; Hct, haematocrit; RR, risk ratio; CI: confidence interval; BMI, Body Mass Index; 1st trimester, first trimester; 2nd trimester, second trimester; 3rd trimester, third trimester; IPW, inverse probability weights; IPCW, inverse probability of censoring weights.

**eTable 6.** Association between weekly change in Hb and Hct levels during pregnancy and risk of stillbirth across different statistical models.

|  | **Adjusted RR (95%CI)** |  |  |
| --- | --- | --- | --- |
|  | **Model1^a^** | **Model2^b^** | **Model3^c^** |
| Weekly change in Hb from 1st trimester to 2nd trimester, g/L | | | |
| <-2.88 (Haemodilution) | 1.83 (1.14, 2.96) | 1.83 (1.12, 2.99) | 1.79 (1.10, 2.90) |
| -2.88 to 0.73 (Reference group) | 1.00 (Reference) | 1.00 (Reference) | 1.00 (Reference) |
| ≥0.74 (Haemoconcentration) | 3.28 (2.04, 5.29) | 3.11 (1.93, 5.00) | 3.16 (1.96, 5.10) |
| Continuous | 1.19 (1.00, 1.42) | 1.22 (1.01, 1.47) | 1.20 (1.00, 1.45) |
| Weekly change in Hb from 1st trimester to 3rd trimester, g/L | | | |
| <-1.48 (Haemodilution) | 0.92 (0.28, 3.00) | 1.00 (0.30, 3.40) | 1.01 (0.30, 3.42) |
| -1.48 to 0.44 (Reference group) | 1.00 (Reference) | 1.00 (Reference) | 1.00 (Reference) |
| ≥0.45 (Haemoconcentration) | 2.17 (0.83, 5.67) | 2.19 (0.85, 5.65) | 2.14 (0.83, 5.51) |
| Continuous | 1.38 (0.83, 2.30) | 1.47 (0.84, 2.56) | 1.43 (0.83, 2.46) |
| Weekly change in Hb from 2nd trimester to 3rd trimester, g/L | | | |
| <-1.72 (Haemodilution) | 1.23 (0.31, 4.99) | 1.71 (0.42, 6.95) | 1.82 (0.44, 7.60) |
| -1.72 to 1.80 (Reference group) | 1.00 (Reference) | 1.00 (Reference) | 1.00 (Reference) |
| ≥1.81 (Haemoconcentration) | 1.76 (0.75, 4.11) | 1.81 (0.76, 4.32) | 1.78 (0.75, 4.22) |
| Continuous | 0.97 (0.75, 1.25) | 0.92 (0.69, 1.23) | 0.92 (0.68, 1.23) |
| Weekly change in Hct from 1st trimester to 2nd trimester, % | | | |
| <-0.83 (Haemodilution) | 1.84 (1.16, 2.89) | 1.78 (1.12, 2.82) | 1.80 (1.14, 2.85) |
| -0.83 to 0.24 (Reference group) | 1.00 (Reference) | 1.00 (Reference) | 1.00 (Reference) |
| ≥0.25 (Haemoconcentration) | 2.85 (1.66, 4.90) | 2.85 (1.64, 4.94) | 2.95 (1.69, 5.15) |
| Continuous | 1.43 (0.73, 2.81) | 1.50 (0.71, 3.14) | 1.45 (0.71, 2.99) |
| Weekly change in Hct from 1st trimester to 3rd trimester, % | | | |
| <-0.40 (Haemodilution) | 1.07 (0.35, 3.32) | 0.96 (0.31, 2.98) | 1.00 (0.32, 3.10) |
| -0.40 to 0.13 (Reference group) | 1.00 (Reference) | 1.00 (Reference) | 1.00 (Reference) |
| ≥0.14 (Haemoconcentration) | 2.39 (0.97, 5.89) | 2.37 (0.97, 5.83) | 2.28 (0.93, 5.59) |
| Continuous | 3.10 (0.47, 20.25) | 4.07 (0.53, 31.07) | 3.43 (0.48, 24.62) |
| Weekly change in Hct from 2nd trimester to 3rd trimester, % | | | |
| <-0.48 (Haemodilution) | 1.41 (0.35, 5.71) | 1.94 (0.48, 7.86) | 2.07 (0.50, 8.62) |
| -0.48 to 0.53 (Reference group) | 1.00 (Reference) | 1.00 (Reference) | 1.00 (Reference) |
| ≥0.54 (Haemoconcentration) | 2.00 (0.86, 4.64) | 2.05 (0.87, 4.83) | 2.02 (0.86, 4.73) |
| Continuous | 1.04 (0.41, 2.67) | 0.89 (0.31, 2.58) | 0.84 (0.27, 2.59) |

Data are n (%) or RR (95% CI). All models were adjusted for maternal age, BMI, gravidity, drinking, region, ethnicity, annual family income, mode of conception, plurality, folic acid use, multivitamin use, medication use in 1st trimester, pre-existing diseases, gestational age at first Hb/Hct measurement. ^a^Relative risk pooled from 50 imputed data sets. ^b^Relative risk pooled from 50 imputed data sets, weighted by IPW to account for exclude group without Hb/Hct values. ^c^Relative risk pooled from 50 imputed data sets, weighted by IPCW to account for loss to follow-up. Abbreviations: Hb, haemoglobin; Hct, haematocrit; RR, risk ratio; CI: confidence interval; BMI, Body Mass Index; 1st trimester, first trimester; 2nd trimester, second trimester; 3rd trimester, third trimester; IPW, inverse probability weights; IPCW, inverse probability of censoring weights.

**eTable 7.** Subgroup analysis of stillbirth risk associated with Hb/Hct levels for singleton and multiple pregnancies.

|  | **Adjusted RR (95%CI) ^a^** | |  |
| --- | --- | --- | --- |
|  | **Singleton pregnancies** | **Multiple pregnancies** | **RERI (95%CI)** |
| Hb in 1st trimester, g/L |  |  |  |
| <70 (Severe anaemia) | 21.47 (3.29, 140.05) | - | - |
| 70-99 (Moderate anaemia) | 2.67 (1.37, 5.19) | - | - |
| 100-109 (Mild anaemia) | 1.21 (0.70, 2.09) | 1.52 (0.35, 6.57) | 0.73 (-4.86, 8.33) |
| 110-129 (Normal Hb) | 1.00 (Reference) | 1.00 (Reference) |  |
| 130-144 (Mildly elevated Hb) | 0.97 (0.79, 1.19) | 1.40 (0.80, 2.43) | 1.68 (-0.78, 4.53) |
| ≥145 (Markedly elevated Hb) | 1.58 (1.04, 2.41) | 1.65 (0.62, 4.41) | 1.93 (-3.14, 9.18) |
| Continuous | 1.00 (0.99, 1.01) | 1.01 (0.99, 1.04) |  |
| Hb in 2nd trimester, g/L |  |  |  |
| <70 (Severe anaemia) | - | - | - |
| 70-94 (Moderate anaemia) | 1.30 (0.53, 3.15) | - | - |
| 95-104 (Mild anaemia) | 1.18 (0.78, 1.79) | 1.34 (0.62, 2.92) | 0.99 (-3.14, 6.15) |
| 105-129 (Normal Hb) | 1.00 (Reference) | 1.00 (Reference) |  |
| 130-144 (Mildly elevated Hb) | 1.29 (0.95, 1.75) | 1.17 (0.52, 2.64) | 0.71 (-3.84, 5.86) |
| ≥145 (Markedly elevated Hb) | 4.19 (1.73, 10.11) | 7.45 (1.53, 36.30) | 29.00 (-11.86, 93.72) |
| Continuous | 1.01 (1.00, 1.02) | 1.01 (0.99, 1.04) |  |
| Hb in 3rd trimester, g/L |  |  |  |
| <70 (Severe anaemia) | - | - | - |
| 70-99 (Moderate anaemia) | 0.56 (0.17, 1.79) | - | - |
| 100-109 (Mild anaemia) | 1.05 (0.64, 1.72) | 2.14 (0.73, 6.24) | 4.52 (-5.65, 17.26) |
| 110-129 (Normal Hb) | 1.00 (Reference) | 1.00 (Reference) |  |
| 130-144 (Mildly elevated Hb) | 2.23 (1.32, 3.77) | 1.86 (0.50, 6.95) | 3.73 (-8.99, 21.63) |
|  |  |  |  |
| ≥145 (Markedly elevated Hb) | - | - | - |
| Continuous | 1.03 (1.00, 1.05) | 1.01 (0.97, 1.06) |  |
| Hct in 1st trimester, % |  |  |  |
| <33 (Anaemia) | 1.70 (1.14, 2.54) | 1.36 (0.49, 3.76) | 0.52 (-4.78, 6.53) |
| 33-39 (Normal Hct) | 1.00 (Reference) | 1.00 (Reference) |  |
| 40-42 (Mildly elevated Hct) | 0.84 (0.64, 1.11) | 0.81 (0.38, 1.72) | -0.68 (-3.53, 2.31) |
| ≥43 (Markedly elevated Hct) | 1.70 (1.03, 2.80) | 1.62 (0.51, 5.15) | 2.13 (-5.07, 12.09) |
| Continuous | 0.99 (0.95, 1.03) | 1.02 (0.92, 1.12) |  |
| Hct in 2nd trimester, % |  |  |  |
| <32 (Anaemia) | 1.07 (0.78, 1.45) | 1.15 (0.62, 2.12) | 0.56 (-2.35, 4.00) |
| 32-39 (Normal Hct) | 1.00 (Reference) | 1.00 (Reference) |  |
| 40-42 (Mildly elevated Hct) | 1.94 (1.18, 3.18) | 1.63 (0.43, 6.19) | 2.06 (-6.79, 13.74) |
| ≥43 (Markedly elevated Hct) | 6.65 (2.44, 18.16) | - | - |
| Continuous | 1.03 (0.99, 1.08) | 1.01 (0.92, 1.11) |  |
| Hct in 3rd trimester, % |  |  |  |
| <33 (Anaemia) | 0.91 (0.56, 1.49) | 0.68 (0.21, 2.22) | -2.81 (-9.96, 5.41) |
| 33-39 (Normal Hct) | 1.00 (Reference) | 1.00 (Reference) |  |
| 40-42 (Mildly elevated Hct) | 1.10 (0.41, 2.95) | - | - |
| ≥43 (Markedly elevated Hct) | 6.85 (1.66, 28.21) | - | - |
| Continuous | 1.07 (0.98, 1.16) | 1.05 (0.91, 1.20) |  |

Data are RR (95% CI). ^a^Associations were adjusted for maternal age, BMI, gravidity, drinking, region, ethnicity, annual family income, mode of conception, folic acid use, multivitamin use, medication use in 1st trimester, pre-existing diseases, and gestational age at Hb/Hct measurement. Abbreviations: RR, risk ratio; CI: confidence interval; RERI, relative excess risk due to interaction; BMI, Body Mass Index; 1st trimester, first trimester; 2nd trimester, second trimester; 3rd trimester, third trimester.

**eTable 8.** Subgroup analysis of stillbirth risk associated with Hb/Hct levels for various trimesters.

|  | **Adjusted RR (95%CI) ^a^** | |  | **Adjusted RR (95%CI) ^a^** | |
| --- | --- | --- | --- | --- | --- |
|  | **Preterm stillbirth** | **Term stillbirth** |  | **SGA stillbirth** | **Non-SGA stillbirth** |
| Hb in 1st trimester, g/L |  |  |  |  |  |
| <70 (Severe anaemia) | 6.82 (1.20, 38.70) | - |  | 41.69 (10.46, 166.18) | - |
| 70-99 (Moderate anaemia) | 2.03 (1.09, 3.78) | - |  | 3.53 (0.89, 13.95) | 2.65 (0.97, 7.24) |
| 100-109 (Mild anaemia) | 1.21 (0.74, 1.98) | - |  | 1.23 (0.29, 5.19) | 1.55 (0.75, 3.24) |
| 110-129 (Normal Hb) | 1.00 (Reference) | 1.00 (Reference) |  | 1.00 (Reference) | 1.00 (Reference) |
| 130-144 (Mildly elevated Hb) | 0.89 (0.73, 1.08) | 2.13 (1.08, 4.22) |  | 0.88 (0.45, 1.70) | 1.04 (0.76, 1.44) |
| ≥145 (Markedly elevated Hb) | 1.30 (0.88, 1.93) | 2.53 (0.68, 9.46) |  | 1.70 (0.54, 5.40) | 1.77 (0.91, 3.44) |
| Continuous | 0.99 (0.98, 1.00) | 1.04 (1.01, 1.07) |  | 0.98 (0.95, 1.01) | 1.00 (0.98, 1.01) |
| Hb in 2nd trimester, g/L |  |  |  |  |  |
| <70 (Severe anaemia) | - | - |  | - | - |
| 70-94 (Moderate anaemia) | 0.96 (0.41, 2.26) | - |  | - | 1.88 (0.68, 5.14) |
| 95-104 (Mild anaemia) | 1.38 (0.97, 1.97) | 0.24 (0.03, 1.74) |  | 0.57 (0.14, 2.31) | 1.17 (0.66, 2.08) |
| 105-129 (Normal Hb) | 1.00 (Reference) | 1.00 (Reference) |  | 1.00 (Reference) | 1.00 (Reference) |
| 130-144 (Mildly elevated Hb) | 1.08 (0.81, 1.43) | 0.68 (0.21, 2.21) |  | 1.17 (0.52, 2.62) | 1.16 (0.69, 1.94) |
| ≥145 (Markedly elevated Hb) | 2.22 (1.07, 4.64) | - |  | - | - |
| Continuous | 1.00 (0.99, 1.01) | 1.01 (0.99, 1.04) |  | 1.00 (0.97, 1.03) | 1.00 (0.98, 1.02) |
| Hb in 3rd trimester, g/L |  |  |  |  |  |
| <70 (Severe anaemia) | - | - |  | - | - |
| 70-99 (Moderate anaemia) | 0.63 (0.21, 1.92) | - |  | - | 0.66 (0.21, 2.11) |
| 100-109 (Mild anaemia) | 1.27 (0.72, 2.23) | 1.25 (0.61, 2.56) |  | 2.06 (0.74, 5.72) | 0.95 (0.54, 1.67) |
| 110-129 (Normal Hb) | 1.00 (Reference) | 1.00 (Reference) |  | 1.00 (Reference) | 1.00 (Reference) |
| 130-144 (Mildly elevated Hb) | 1.71 (0.95, 3.09) | 1.40 (0.60, 3.28) |  | 2.03 (0.65, 6.31) | 2.02 (1.10, 3.72) |
| ≥145 (Markedly elevated Hb) | - | - |  | - | - |
| Continuous | 1.01 (0.99, 1.04) | 1.02 (0.99, 1.04) |  | 0.99 (0.95, 1.04) | 1.03 (1.00, 1.05) |
| Hct in 1st trimester, % |  |  |  |  |  |
| <33 (Anaemia) | 1.49 (1.03, 2.15) | 0.73 (0.10, 5.32) |  | 2.74 (1.08, 6.95) | 1.52 (0.81, 2.85) |
| 33-39 (Normal Hct) | 1.00 (Reference) | 1.00 (Reference) |  | 1.00 (Reference) | 1.00 (Reference) |
| 40-42 (Mildly elevated Hct) | 0.73 (0.56, 0.95) | 1.32 (0.60, 2.91) |  | 0.77 (0.31, 1.87) | 0.65 (0.41, 1.05) |
| ≥43 (Markedly elevated Hct) | 1.36 (0.86, 2.16) | 1.20 (0.16, 9.15) |  | 1.06 (0.16, 7.19) | 1.83 (0.86, 3.93) |
| Continuous | 0.97 (0.94, 1.01) | 1.11 (1.00, 1.23) |  | 0.91 (0.80, 1.04) | 0.99 (0.94, 1.05) |
| Hct in 2nd trimester, % |  |  |  |  |  |
| <32 (Anaemia) | 1.22 (0.93, 1.62) | 0.62 (0.25, 1.58) |  | 1.60 (0.70, 3.65) | 1.04 (0.67, 1.63) |
| 32-39 (Normal Hct) | 1.00 (Reference) | 1.00 (Reference) |  | 1.00 (Reference) | 1.00 (Reference) |
| 40-42 (Mildly elevated Hct) | 1.50 (0.95, 2.37) | 1.24 (0.17, 8.76) |  | 2.79 (0.93, 8.38) | 1.39 (0.56, 3.42) |
| ≥43 (Markedly elevated Hct) | 4.16 (1.69, 10.22) | - |  | - | 9.74 (2.29, 41.38) |
| Continuous | 1.00 (0.96, 1.04) | 1.01 (0.92, 1.11) |  | 0.99 (0.88, 1.12) | 1.00 (0.94, 1.07) |
| Hct in 3rd trimester, % |  |  |  |  |  |
| <33 (Anaemia) | 0.99 (0.57, 1.71) | 0.89 (0.43, 1.86) |  | 2.06 (0.68, 6.24) | 0.85 (0.49, 1.47) |
| 33-39 (Normal Hct) | 1.00 (Reference) | 1.00 (Reference) |  | 1.00 (Reference) | 1.00 (Reference) |
| 40-42 (Mildly elevated Hct) | 0.46 (0.11, 1.95) | 1.22 (0.32, 4.69) |  | - | 1.40 (0.52, 3.77) |
| ≥43 (Markedly elevated Hct) | 4.70 (1.30, 16.99) | - |  | - | 8.96 (2.18, 36.89) |
| Continuous | 1.01 (0.93, 1.10) | 1.06 (0.96, 1.16) |  | 0.93 (0.78, 1.13) | 1.07 (0.97, 1.17) |

Data are RR (95% CI). ^a^Associations were adjusted for maternal age, BMI, gravidity, drinking, region, ethnicity, annual family income, mode of conception, plurality, folic acid use, multivitamin use, medication use in 1st trimester, pre-existing diseases, and gestational age at Hb/Hct measurement. Abbreviations: RR, risk ratio; CI: confidence interval; SGA, small for gestational age; BMI, Body Mass Index; 1st trimester, first trimester; 2nd trimester, second trimester; 3rd trimester, third trimester.
